# Pathogenicity and Toxigenic Chemotype Analysis of *Fusarium* Species Causing Maize Stalk Rot and Evaluation of *Trichoderma* for Biocontrol and Mycotoxin Suppression

**DOI:** 10.3390/jof12070505

**Published:** 2026-07-09

**Authors:** Nan Chen

**Affiliations:** College of Plant Protection, Gansu Agricultural University, Lanzhou 730070, China; chenn@gsau.edu.cn

**Keywords:** antagonism, inhibition rate, biological control

## Abstract

This study assessed the pathogenicity and toxigenic chemotypes of *Fusarium* species causing maize stalk rot. In addition, the ability of selected strains of *Trichoderma longibrachiatum*, *T. koningiopsis*, and *T. gamsii* to inhibit mycelial growth and the biosynthesis of *Fusarium* mycotoxins was evaluated. Pathogenicity assays indicated that *F. graminearum*, *F. verticillioides*, *F. boothii*, and *F. subglutinans*, previously characterized via morphological and molecular analyses, caused maize stalk rot symptoms with varying degrees of aggressiveness. PCR-based detection of mycotoxin biosynthetic genes revealed distinct toxigenic chemotypes among the *Fusarium* isolates, including fumonisin (FUM), 3-acetyl-deoxynivalenol (3-AcDON), zearalenone (ZEN), and beauvericin (BEA). After 7 days of co-culture on potato dextrose agar, all *Trichoderma* strains significantly inhibited the growth of *Fusarium* mycelia. The *Fusarium* pathogen inhibition rate ranged between 28.30 and 71.77%. *T. longibrachiatum* grew over the mycelium of the pathogen and sporulated. In rice-based co-cultured assays, *Trichoderma* strains inhibit FUM biosynthesis by about 74% to 92%, 3-AcDON by about 32% to 100%, ZEN by about 11% to 90%, and BEA by about 6% to 57%, depending on the pathogen–antagonist combination. The maximum reduction in mycotoxin production (100% for 3-AcDON) exceeded the maximum inhibition of mycelial growth (71.77%), suggesting that *Trichoderma* may repress mycotoxin biosynthesis via mechanisms independent of simple growth inhibition. These findings provide a basis for developing green biocontrol strategies for maize stalk rot.

## 1. Introduction

Maize stalk rot is a typical soil-borne fungal disease that occurs during maize growth [1]. Factors including variety replacement, continuous cropping, and farming system adjustments have led to an increasing incidence of this disease in China’s maize-producing areas [2]. This has severely affected maize yield and quality, becoming a major constraint on achieving high and stable yields. Maize stalk rot is caused by multiple pathogenic fungi, with *Fusarium* spp. being among the most significant causal agents [3,4]. During the infection of maize, *Fusarium* spp. not only cause plant lodging, basal stalk rot, and ear drop but also produce various mycotoxins, primarily deoxynivalenol (DON), zearalenone (ZEN), and fumonisins (FUM) [5,6]. These mycotoxins can persist in maize kernels and stems, posing health risks to humans and livestock through the food chain by inducing adverse effects such as vomiting and immunosuppression [7,8]. Additionally, mycotoxins can disrupt plant cellular structures, inhibit protein synthesis, accelerate wilting, and facilitate pathogen evasion of host defense mechanisms, thereby promoting disease progression and spread and significantly increasing the difficulty of preventing and controlling maize stalk rot [9].

Currently, the prevention and control of maize stalk rot in agricultural production relies primarily on the application of chemical agents combined with the removal of diseased plant residues from the field [10]. However, chemical agents cannot degrade *Fusarium* mycotoxins, and their long-term, large-scale application can easily lead to pesticide residues and environmental pollution [11]. As the requirements for green and sustainable agricultural development continue to increase, the limitations of existing prevention and control strategies are becoming increasingly evident [12]. Recently, the potential of microorganisms exhibiting antagonistic properties against various pathogens has received increasing attention. Plant disease control using microorganisms offers several advantages, as these not only compete with pathogens but also enhance plant performance through their biostimulatory action [13,14]. Of particular interest among potential biocontrol microorganisms are species in the genus of *Trichoderma* [15]. *Trichoderma* spp. exhibit antagonistic properties and diverse biostimulatory activities in plants [16]. These include competing for space and nutrients, mycoparasitism, and antimicrobial effects [17]. Many *Trichoderma* spp. have been used as agents to enhance the nutrient use efficiency of crops [18].

Recent studies have shown that *Trichoderma* spp. not only display antagonistic properties toward other pathogenic fungi but also inhibit the biosynthesis of mycotoxins [19]. Furthermore, enzymes secreted by these fungi may promote mycotoxin degradation or biotransformation [20]. Biocontrol measures remain relatively underexplored in research on the prevention and control of maize stalk rot [21]. Therefore, pre-selecting different strains of individual *Trichoderma* spp. to assess their antagonistic potential is necessary. In this study, the pathogenicity and toxigenic chemotypes of *Fusarium* spp. causing maize stalk rot were determined. In addition, the antagonistic effects of *Trichoderma* against these pathogens and its ability to inhibit *Fusarium* mycotoxin biosynthesis were evaluated.

## 2. Materials and Methods

### 2.1. Fungal Strains

Four strains of *Fusarium* spp. (i.e., *F. graminearum* GS2401, *TEF-1α* accession number PZ580594; *F. verticillioides* GS2403, *TEF-1α* accession number PZ580593; *F. boothii* GS2407, *TEF-1α* accession number PZ580598; and *F. subglutinans* GS2410, *TEF-1α* accession number PZ580592) and three strains of *Trichoderma* spp. (i.e., *T. longibrachiatum* TL2501, ITS accession number PZ567814, *tef1-α* accession number PZ580596; *T. koningiopsis* TK2502, ITS accession number PZ571067, *tef1-α* accession number PZ580595; and *T. gamsii* TG2503, ITS accession number PZ571075, *tef1-α* accession number PZ580597) were used in this study. All strains had been previously identified based on morphological characteristics and molecular analyses. For molecular identification, the internal transcribed spacer (ITS) region (primers ITS1/ITS4) and the translation elongation factor 1α (*tef1-α*) gene (primers EF1-728/TEF1LLErev) were amplified and sequenced for *Trichoderma* strains, whereas *Fusarium* species were identified by sequencing the translation elongation factor 1α (*TEF-1α*) gene (primers EF-1/EF-2). The *Fusarium* species are dominant pathogens of maize stalk rot with high isolation frequency in fields. All strains were deposited in the College of Plant Protection, Gansu Agricultural University, China.

### 2.2. Pathogenicity Test on Maize Stalks

Zhengdan958, one of the most widely cultivated maize cultivars in China, was selected as the host material for the pathogenicity assay in this study. At the 10-leaf stage, Zhengdan958 plants were inoculated by punching a hole in the stalk at the second or third internode above the soil line using a sterile toothpick. Thereafter, 25 μL conidia suspension from each tested *Fusarium* strain was injected at a concentration of 10^6^ mL^−1^. Maize stalks treated with sterile water served as the mock-inoculation control. The inoculation site was wrapped with sterilized gauze to maintain moisture and prevent contamination. Each treatment contained three biological replicates, with three plants inoculated per replicate. Seven days post-inoculation (dpi), the stalks of inoculated maize plants were bisected longitudinally to evaluate disease symptoms. The longitudinal brown necrotic lesions were measured as the necrotic area to quantify the virulence of each identified *Fusarium* species using ImageJ software (Version 1.54p, National Institutes of Health, Bethesda, MD, USA) [22,23].

### 2.3. Genomic DNA Extraction

All tested *Fusarium* strains were grown on PDA medium (Oxoid, Basingstoke, UK) and incubated for 7 days at 25 °C. Mycelium was recovered with a sterile spatula, and total genomic DNA was extracted using a Fungal Genomic DNA Kit (TIANGEN, Beijing, China) according to the manufacturer’s instructions. DNA concentration and purity were determined using a NanoDrop spectrophotometer (Thermo Fisher Scientific, Waltham, MA, USA), and DNA quality was assessed using 1.0% agarose gel electrophoresis [24]. The extracted DNA was stored at −20 °C until use for PCR-based toxigenic chemotype identification.

### 2.4. Determination of Fusarium Toxigenic Chemotypes

To determine the toxigenic chemotypes of the four tested *Fusarium* strains, four mycotoxin biosynthesis-related genes (*FUM5*, *esyn1*, *PKS4*, and *Tri13*) were amplified by PCR using specific primers as previously described [22,25,26,27,28]. The sequences of primers used to amplify these genes are listed in Table 1. PCR was conducted in a 20-μL reaction mixture comprising 1 μL template DNA, 10 μL of 2× Premix Taq PCR Mix (Takara Bio Inc., Shiga, Japan), 8 μL sterile water, and 0.5 μL of each primer (10 μM). Amplification reactions were performed in a C1000 Touch thermal cycler (Bio-Rad Laboratories, Hercules, CA, USA). Nuclease-free sterile water was included as a negative control in the PCR reaction to exclude potential reagent or environmental contamination.

### 2.5. Dual-Culture Bioassay

Experiments to evaluate the inhibition of pathogenic *Fusarium* growth were conducted in co-cultures on PDA medium (Oxoid) [29]. The fungal culture was grown on 9.0 cm diameter Petri dishes. The antagonistic fungi and pathogenic *Fusarium* were simultaneously inoculated on opposite sides of the plate using 8 mm diameter plugs excised from the actively growing margins of 7-day-old cultures, maintaining a distance of approximately 6.0 cm between plugs. Single cultures on PDA plates represented control samples. Single-strain controls were positioned consistently with matched dual cultures to eliminate position-related growth bias. Plates were incubated at 25 ± 2 °C under a 12 h light/12 h dark photoperiod. After 7 days of incubation, the diameter of *Fusarium* colonies was measured. The experiment was conducted in independent triplicates.

After 7 days of co-incubation, a qualitative assessment of pathogen–antagonist interactions was conducted using the scale proposed by Mańka [30]. The scale shows the degree of domination of one colony by another and takes the following values: 0 (the antagonist mycelium has outgrown 50% of the plate surface area), +4 (the antagonist mycelium has outgrown 75% of the plate surface area), +6 (the antagonist mycelium has outgrown 85% of the plate surface area), +8 (the antagonist mycelium has outgrown 95% of the plate surface area and/or the pathogen mycelium).

Growth inhibition of pathogenic *Fusarium* by *Trichoderma* isolates was determined by calculating the percentage reduction in pathogen radial growth using the following formula: (*Rc* − *R*)/*Rc* × 100%, where *Rc* is the radial growth of the pathogen in the control sample and *R* is the radial growth of the pathogen in the co-culture [29]. Radial growth was measured using a digital caliper and expressed in millimeters (mm). All experiments were performed in three biological replicates, and data are presented as mean ± standard deviation (SD).

### 2.6. Determination of Mycotoxin Biosynthesis Inhibition

To determine the inhibition of mycotoxin biosynthesis in *Fusarium* by *Trichoderma*, they were simultaneously inoculated on rice grains. The control samples comprised *Fusarium* strains growing in single cultures. Thirty grams of long-grain white rice and 8 mL of sterile water were added to an Erlenmeyer flask, incubated overnight, and subsequently sterilized by autoclaving at 121 °C for 30 min on the following day [31]. Next, the rice was inoculated with 3 cm^2^ of 7-day-old mycelium grown on PDA medium (Oxoid) [31]. The average culture humidity was maintained at approximately 30% for 14 days. After incubation, the cultures were dried at 50 ± 2 °C and ground. Afterward, mycotoxin levels were quantified using four commercial ELISA kits, including fumonisin (FUM; Cat. No. JM-0669101), 3-Ac-deoxynivalenol (3-AcDON; Cat. No. JM-0317Z1), zearalenone (ZEN; Cat. No. JM-0662501), and beauvericin (BEA; Cat. No. JM-0024N1) (Jingmei Biotechnology Co., Ltd., Yancheng, Jiangsu, China), according to the manufacturer’s instructions.

### 2.7. Statistical Analysis

For the statistical analysis, SPSS software (Version 26.0, SPSS Inc., Chicago, IL, USA) was used to calculate the average value and analyze the variance of the test data, while Duncan’s new multiple range test was applied for multiple comparisons (*p* ≤ 0.05).

## 3. Results

### 3.1. Pathogenicity Tests on Maize Stalks

**Figure 1:**
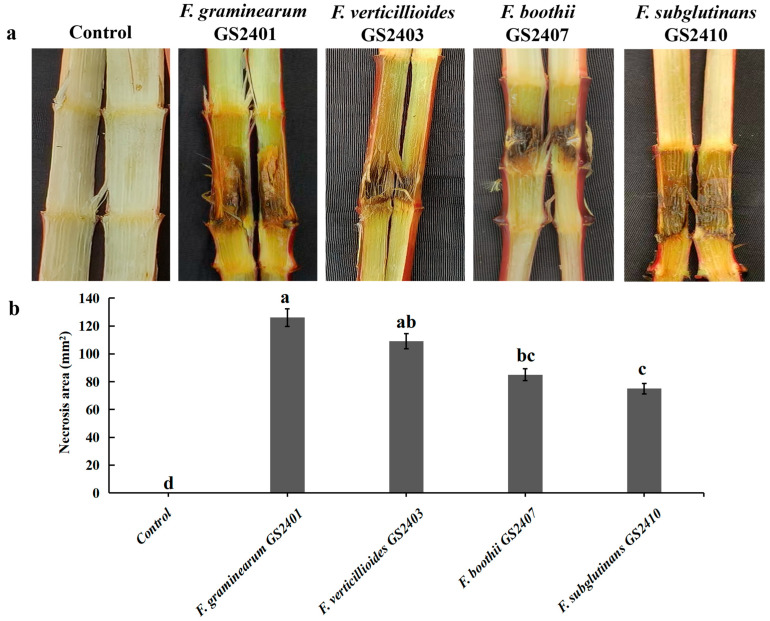
Symptoms on maize stalks inoculated with *Fusarium* isolates. (**a**) Symptoms of maize stalks inoculated with *Fusarium* isolates at 7 dpi. No symptoms were observed in the control plants. (**b**) Statistical analysis of necrotic areas around the insertion point inoculated with *Fusarium* isolates at 7 dpi. The longitudinal brown infected areas were used to evaluate the virulence of each identified *Fusarium* species. Each assay was performed using three independent biological replicates. Different lowercase letters indicate significant pathogenicity differences among *Fusarium* spp. at *p* < 0.05, as determined by Duncan’s multiple range test.

To assay the pathogenicity of the four tested *Fusarium* strains, the stalks of Zhengdan958 maize plants at the 10-leaf stage were inoculated with each strain. The symptoms and severity of the disease were recorded at 7 dpi. The results showed that all tested *Fusarium* spp. were pathogenic to maize stalks and caused distinct discoloration of internal stalk tissues around the inoculation site (Figure 1a). The longitudinal brown infected areas were measured to evaluate the virulence of each identified *Fusarium* species (Figure 1b). *Fusarium graminearum* was the most aggressive among all tested *Fusarium* strains. The pathogenicity of these strains was confirmed by reisolating the fungi from symptomatic tissues but not from the control plants.

### 3.2. Analysis of Fusarium Toxigenic Chemotypes

Molecular detection of toxigenic chemotypes in tested *Fusarium* strains was performed using PCR-based methods. The primer pair Tri13P1/2 was used to amplify specific DNA fragments of 583 bp for 15-AcDON, 644 bp for 3-AcDON, and 859 bp for NIV. In addition, the *FUM5* gene was used to detect FUM-producing strains, yielding an amplicon of 845 bp. The *PKS4* gene was selected to detect ZEN-producing strains, generating a 280 bp fragment, while the *esyn1* gene was used to identify BEA-producing strains, yielding a 600 bp amplicon.

**Figure 2:**
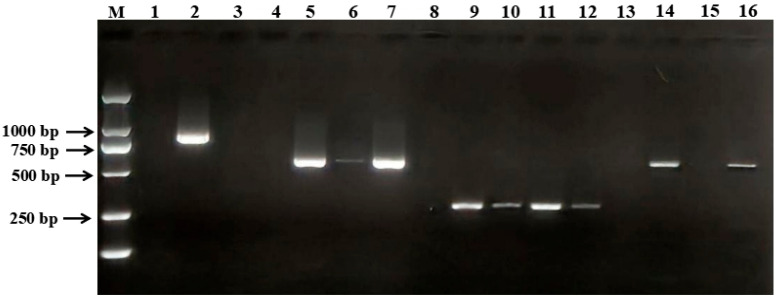
PCR-based analysis of toxigenic chemotypes in four *Fusarium* species. Lane M: marker; Lanes 1–4: detection of fumonisin biosynthetic gene (*FUM5*) in *F. graminearum*, *F. verticillioides*, *F. boothii*, and *F. subglutinans*, respectively; Lanes 5–8: *Tri13*-based detection of trichothecene chemotypes (3-AcDON, 15-AcDON and nivalenol-producing types) in *F. graminearum*, *F. verticillioides*, *F. boothii*, and *F. subglutinans*, respectively; Lanes 9–12: detection of zearalenone biosynthetic gene (*PKS4*) in *F. graminearum*, *F. verticillioides*, *F. boothii*, and *F. subglutinans*, respectively; Lanes 13–16: detection of beauvericin biosynthetic gene (*esyn1*) in *F. graminearum*, *F. verticillioides*, *F. boothii*, and *F. subglutinans*, respectively.

The PCR amplification results revealed that all four tested *Fusarium* species possessed the genetic potential to produce mycotoxins; the amplification profiles of these strains are presented in Figure 2. Among the four *Fusarium* strains, *F. graminearum* harbored genes for 3-Ac-deoxynivalenol (3-AcDON) and ZEN. *F. verticillioides* contained biosynthetic genes for FUM, 3-AcDON, ZEN, and BEA. *F. boothii* carried genes encoding 3-AcDON and ZEN production, while *F. subglutinans* produced ZEN and BEA (Figure 2).

### 3.3. Evaluation of the Antagonistic Potential of Trichoderma spp.

**Figure 3:**
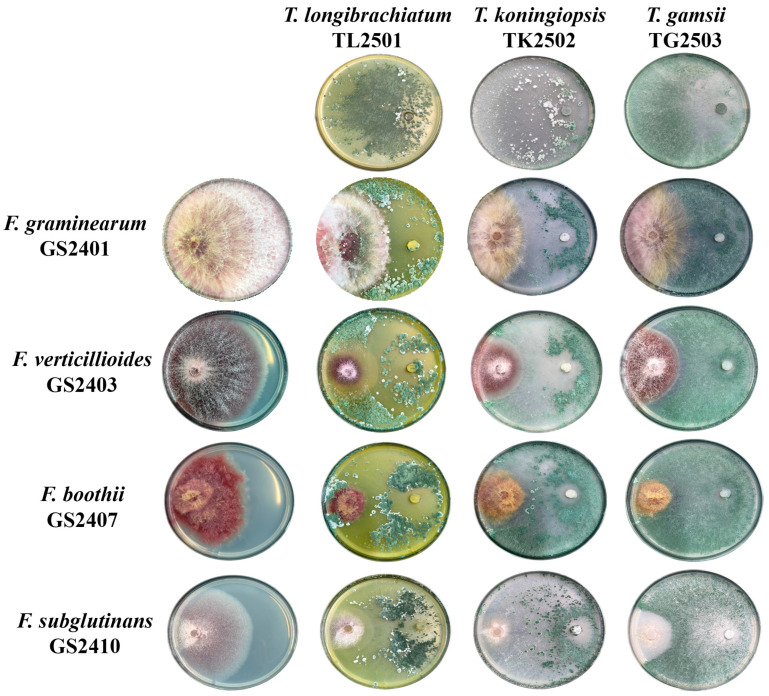
Fungal morphology of *Fusarium* spp. in a dual culture assay on PDA after 7 days of incubation. *F. graminearum* GS2401; *F. verticillioides* GS2403; *F. boothii* GS2407; *F. subglutinans* GS2410. *Fusarium* strain and *Trichoderma* strain grown alone control, *Fusarium* strains with *T. longibrachiatum*, *T. koningiopsis*, and *T. gamsii*.

Antagonistic tests showed that all *Trichoderma* strains inhibited the growth of the tested *Fusarium* strains (Figure 3). After 7 days of incubation, the pathogen inhibition rate ranged between 28.30 and 71.77%, depending on the pathogen and the *Trichoderma* strain (Table 2). Among them, *T. koningiopsis* TK2502 exhibited the strongest growth inhibition against *F. graminearum* GS2401 and *F. subglutinans* GS2410, with inhibition rates of 31.46% and 71.77%, respectively. *T. longibrachiatum* TL2501 showed the highest inhibition rate of 54.63% against *F. verticillioides* GS2403, whereas *T. gamsii* TG2503 exhibited the strongest inhibitory effect against *F. boothii* GS2407, with an inhibition rate of 63.53% (Table 2). Overall, *T. koningiopsis* TK2502 displayed the broadest antagonistic activity among the three *Trichoderma* strains. *F. subglutinans* was the most susceptible to *Trichoderma* spp. inhibition, whereas *F. graminearum* was the most resistant.

After 7 days of *Trichoderma* spp. and *Fusarium* spp. co-culture, a qualitative assessment was performed using the Mańka scale (Table 3). The Mańka scale quantifies the antagonistic potential of *Trichoderma* spp., with higher scores indicating stronger suppression of pathogen hyphae. Both *T. longibrachiatum* TL2501 and *T. gamsii* TG2503 exhibited antagonistic activity against *F. verticillioides* GS2403, *F. boothii* GS2407, and *F. subglutinans* GS2410, with all co-culture pairs attaining a score of 6. This score denotes that antagonist mycelia overgrew 85% of the plate surface area, revealing their prominent inhibitory capacity against the three aforementioned *Fusarium* strains. In contrast, both isolates received a score of 0 when challenged with *F. graminearum* GS2401, under which condition antagonist mycelia merely covered 50% of the plate surface area. This score illustrates that the two *Trichoderma* strains displayed markedly weaker suppression against *F. graminearum* GS2401 relative to the other three *Fusarium* spp. (Table 3). Similarly, *T. koningiopsis* TK2502 also attained a score of 6 against the three aforementioned *Fusarium* strains, yet only scored 4 against *F. graminearum* GS2401, with its hyphae covering 75% of the plate surface area. This result suggests that its inhibitory effect on *F. graminearum* GS2401 was weaker than that on the other three *Fusarium* spp. (Table 3). Overall, among all tested *Trichoderma* spp. in the qualitative assessment, *T. koningiopsis* TK2502 received the highest average score of 5.5 on the Mańka scale (Table 3). *T. longibrachiatum* TL2501 outgrew and sporulated on the mycelium of all studied *Fusarium* strains. *Trichoderma koningiopsis* and *T. gamsii* also overgrew the mycelium of all studied *Fusarium* strains (Figure 3). Collectively, these results further confirmed that *T. koningiopsis* TK2502 exhibited the optimal inhibitory activity against the tested *Fusarium* spp.

### 3.4. Effect of Trichoderma spp. on Mycotoxin Biosynthesis by Fusarium spp.

The ability of *Trichoderma* spp. to inhibit the biosynthesis of *Fusarium* mycotoxins was assessed. FUM, 3-AcDON, ZEN, and BEA were present in the post-culture media (Table 4). 3-AcDON and ZEN were identified in the control *F. graminearum* GS2401 at levels of 4.97 and 296.61 ng mL^−1^, respectively. The levels of these toxins in the co-cultures were lower than those in the control medium, ranging from 0.61 to 2.59 ng mL^−1^ for 3-AcDON and 46.98 to 104.36 ng mL^−1^ for ZEN. FUS and BEA were not detected in either the control or co-culture medium (Table 4).

*Fusarium verticillioides* GS2403 produced FUM, 3-AcDON, ZEN, and BEA in the control culture at concentrations of 6.67, 4.35, 96.30, and 0.49 ng mL^−1^, respectively. The levels of FUM, 3-AcDON, ZEN, and BEA were lower in media from co-culture than in the control sample (*F. verticillioides* GS2403), and ranged from 0.51 to 1.73 ng mL^−1^ for FUM, 0.96 to 1.30 ng mL^−1^ for 3-AcDON, 15.13 to 70.13 ng mL^−1^ for ZEN, and 0.21 to 0.46 ng mL^−1^ for BEA (Table 4).

*Fusarium boothii* GS2407 synthesized 3-AcDON and ZEN in the control culture at concentrations of 0.96 and 58.89 ng mL^−1^, respectively. 3-AcDON was not detected in the co-culture of *F. boothii* GS2407 with *T. longibrachiatum* TL2501, whereas it was detected in other co-culture media at concentrations ranging from 0.51 to 0.65 ng mL^−1^. For ZEN, the levels in the co-culture media were lower than those in the control sample (*F. boothii* GS2407). FUS and BEA were not detected in either the control or co-culture medium (Table 4).

*Fusarium subglutinans* GS2410 strain synthesized ZEN and BEA at 277.37 and 0.33 ng mL^−1^, respectively. In all co-culture combinations, *Thichoderma* strains reduced mycotoxin levels, which ranged from 46.88 to 246.21 ng mL^−1^ for ZEN and 0.24 to 0.26 ng mL^−1^ for BEA. FUS and 3-AcDON were not detected in either the control or co-culture medium (Table 4).

Notably, the *Trichoderma* spp. exerted stronger inhibition on mycotoxin production than on mycelial growth of *Fusarium* spp. in most *Fusarium*–*Trichoderma* combinations. Across all treatments, the maximum inhibition rate of mycotoxin production reached 100%, which was markedly higher than the maximum mycelial growth inhibition rate of 72% (Table 4). This suggests that the reduction in mycotoxin production is not solely due to restricted fungal growth. Instead, *Trichoderma* spp. likely exert specific inhibitory effects on mycotoxin biosynthesis and may also contribute to mycotoxin degradation or adsorption, resulting in stronger suppression of mycotoxin levels than of fungal growth itself.

## 4. Discussion

*Fusarium* spp. cause maize stalk rot at different growth stages and may infect maize via systemic spread following root colonization, through young leaf sheaths, by seed transmission, and through wounds caused by hail or insect feeding [32,33]. Maize stalk rot results in defective grain filling, premature senescence, and lodging, which negatively affect production, harvesting, and yield [5]. In the present study, pathogenicity tests showed that all tested *Fusarium* strains could cause severe maize stalk rot symptoms, but the extent of lesion spread varied among strains. *Fusarium graminearum* was the most aggressive species infecting maize stalks. To investigate the mycotoxin-producing ability of *Fusarium* species causing maize stalk rot, toxigenic chemotypes were evaluated using specific PCR assays. A previous study on *Fusarium* reported that pathogen aggressiveness was related to the type of mycotoxin it produced [34]. In addition, the pathogenicity of *F. graminearun* to wheat plants is reportedly closely associated with the type of mycotoxin produced [35].

*Trichoderma* spp. are among the most aggressive antagonists of *Fusarium*, and their key biocontrol mechanisms include competition for space, antibiosis, and mycoparasitism [36,37]. The present study showed that all tested *Trichoderma* strains were capable of inhibiting *Fusarium* growth, with an average inhibition rate of 28.30–71.77%, depending on the *Trichoderma* strain. To date, published research has demonstrated the potential of *Trichoderma* species to limit the growth of pathogenic *Fusarium* species, and these results are consistent with our findings. Larran et al. [38] showed that *T. harzianum* in a co-culture with *F. graminearum* inhibited pathogenic mycelial growth by 46% in vitro. Xue et al. [39] reported relatively high (up to 81%) in vitro inhibition of *F. graminearum* growth by *T. citrinoviride*, *T. harzianum*, and *T. asperellum* [39]. The discrepancies between our results and published results were probably caused by differences in the experimental models used. Veenstra et al. [40] observed 52% growth inhibition of *F. verticillioides* by *T. asperellum* after simultaneous strain inoculation on PDA medium, likely due to competition for resources, as *T. asperellum* grew faster than *F. verticillioides*.

The studied *Trichoderma* strains inhibited mycotoxin biosynthesis by *Fusarium* fungi (FUM, 3-AcDON, ZEN, and BEA) by 6–100% relative to that in the control samples. This inhibition was likely due to a reduction in pathogenic mycelial growth and/or the interference between *Fusarium* and *Trichoderma* metabolic pathways. Similar studies have shown that co-culturing *F. graminearum* with various *Trichoderma* strains inhibits ZEN biosynthesis by 9–97%, α-zearalenol (ZOL) by 31–87%, and β-ZOL by 34–89% [29,41]. In the current study, mycotoxin biosynthesis was inhibited by 74–92% for FUM, 32–100% for 3-AcDON, 27–90% for ZEN, and 6–57% for BEA. Our findings are consistent with those of Modrzewska et al. [31].

Mycotoxin production by the four *Fusarium* strains and its regulation under *Tricoderma* co-culture were evaluated. In monoculture, *F. graminearum* GS2401 produced 3-AcDON and ZEN, with no FUS or BEA detected. This finding is consistent with Huang et al. [42], who indicated that *F. graminearum* primarily produces 3-AcDON and ZEN but not FUS/BEA. Notably, this conclusion is limited to the single *F. graminearum* isolate used in the present study. Meanwhile, co-culture with *Trichoderma* significantly reduced both toxins, aligning with the inhibitory direction observed previously [43]. However, the extent of inhibition varied, with reported inhibition rates reaching up to 99.4% for 3-AcDON produced by *F. graminearum* [43]. Tian et al. [41] suggested ZEN sulfation as one of the ZEN detoxification processes by *Trichoderma* after cultivating cultures of *T. harzianum*, *T. koningii*, *T. longibrachiatum*, *T. atroviride*, *T. asperellum*, and *T. virens* on a ZEN-containing medium. In addition, *Trichoderma* inhibited all four toxins produced by *F. verticillioides* GS2403, although the inhibitory efficiency varied among mycotoxins. Among them, 3-AcDON exhibited the greatest reduction (up to 100%), while BEA showed the smallest reduction (only 6%). Reported inhibition rates for *Trichoderma* include up to 99.4% for 3-AcDON, 12–100% for ZEN, and up to 98% for FUM, with no significant effect observed on BEA [43,44]. In the present study, *Trichoderma* inhibited 3-AcDON synthesis by *F. boothii* GS2407 and also reduced ZEN production. *Trichoderma longibrachiatum* TL2501 completely inhibited 3-AcDON synthesis by *F. boothii* GS2407. *Trichoderma* also exhibited inhibitory effects against ZEN and BEA produced by *F. subglutinans* GS2410. The current study demonstrated that *Trichoderma* exhibits inhibitory effects on mycotoxins produced by various *Fusarium* species; however, the magnitude of inhibition varies depending on the toxin type and *Fusarium* strain.

The mechanism underlying mycotoxin reduction by *Trichoderma* spp. has been debated. Some studies suggest that *Trichoderma* spp. directly inhibit mycotoxin biosynthesis through suppression of mycotoxin biosynthetic gene expression [45,46]. In the present study, the mycotoxin inhibition rates consistently exceeded the mycelial growth inhibition rates for most *Fusarium*–*Trichoderma* combinations. For example, *T. koningiopsis* TK2502 reduced *F. graminearum* growth by only 31% but decreased 3-AcDON and ZEN production by 88% and 84%, respectively. This disparity between growth inhibition and mycotoxin inhibition suggests that *Trichoderma* may interfere with the expression or activity of key enzymes involved in the mycotoxin biosynthetic pathways.

While *Trichoderma* has shown promising antagonistic potential against *Fusarium* causing maize stalk rot, this study has several limitations. All experiments were conducted in vitro, which does not fully represent field environments. Moreover, the limited number of tested strains restricted the generalizability of the results. The molecular mechanisms underlying the antagonistic activity and mycotoxin inhibition of *Trichoderma* remain unclear, particularly its weak inhibitory effect on BEA, which was only 6%. Additionally, the long-term colonization ability of *Trichoderma* was not evaluated. Future research will conduct large-scale field trials to verify the biocontrol efficacy of *Trichoderma*, reveal the interaction mechanism between *Trichoderma* and *Fusarium* using multi-omics technologies, and genetically engineer *Trichoderma* to improve its mycotoxin inhibition capacity, aiming to provide further support for the green and sustainable control of maize stalk rot.

## Figures and Tables

**Table 1:** Sequences of primer pairs used to detect mycotoxin-coding genes in *Fusarium* species.

Mycotoxin	Gene	Primers	Primers Sequence (5′–3′)	Tm (°C)	Amplification Size (bp)	References
Fumonisin	*FUM5*	Fum5F	GTCGAGTTGTTGACCACTGCG	62	845	[25]
		Fum5R	CGTATCGTCAGCATGATGTAGC			
Beauvericin	*esyn1*	esy1	TTCAAGGGCTGGACGTCTATGTA	58	600	[26]
		esy2	GTGAAGAAAGCTGGCTCAACGAG			
Zearalenone	*PKS4*	F1	CGTCTTCGAGAAGATGACAT	58	280	[27]
		R1	TGTTCTGCAAGCACTCCGA			
Nivalenol	*Tri13*	Tri13P1	CTCSACCGCATCGAAGASTCTC	58	859	[28]
		Tri13P2	GAASGTCGCARGACCTTGTTTC			
3-AcDON	*Tri13*	Tri13P1	CTCSACCGCATCGAAGASTCTC	58	644	[28]
		Tri13P2	GAASGTCGCARGACCTTGTTTC			
15-AcDON	*Tri13*	Tri13P1	CTCSACCGCATCGAAGASTCTC	58	583	[28]
		Tri13P2	GAASGTCGCARGACCTTGTTTC			

**Table 2:** Inhibition of mycelial growth of *Fusarium* spp. by *Trichoderma* spp. after 7 days of co-culture on PDA medium.

Pathogen/ Antagonist	*F. graminearum* GS2401	*F. verticillioides* GS2403	*F. boothii* GS2407	*F. subglutinans* GS2410
*T. longibrachiatum* TL2501	28.30 ± 4.40 a	54.63 ± 3.53 a	60.13 ± 3.37 a	67.57 ± 5.25 a
*T. koningiopsis* TK2502	31.46 ± 1.59 a	52.43 ± 1.66 a	57.47 ± 12.95 a	71.77 ± 6.01 a
*T. gamsii* TG2503	29.47 ± 2.72 a	46.57 ± 0.61 b	63.53 ± 3.70 a	65.53 ± 4.21 a

Data are presented as mean ± standard deviation (SD). Different lowercase letters within the same column indicate significant differences in the inhibition of mycelial growth of *Fusarium* spp. among *Trichoderma* spp. (*p* < 0.05), as determined by Duncan’s multiple range test.

**Table 3:** The assessment of the interaction between *Trichoderma* spp. vs. *Fusarium* spp. colonies after 7 days of co-culture on PDA medium.

Pathogen/ Antagonist	*F. graminearum* GS2401	*F. verticillioides* GS2403	*F. boothii* GS2407	*F. subglutinans* GS2410	Average *
*T. longibrachiatum* TL2501	0	6	6	6	4.5
*T. koningiopsis* TK2502	4	6	6	6	5.5
*T. gamsii* TG2503	0	6	6	6	4.5

*—Average Mańka scale score calculated across the four tested Fusarium strains for each Trichoderma isolate; identical scores were obtained from all triplicate replicate plates for each antagonist–pathogen pairing.

**Table 4:** Mycelial growth inhibition on PDA medium and mycotoxin production of *Fusarium* strains cultured on rice kernels with or without *Trichoderma* strains.

Strain	Mycotoxin (ng mL^−1^)				Mycelial Growth (mm)
	FUS	3-AcDON	ZEN	BEA	
*F. graminearum* GS2401 control	ND	4.97 ± 2.14 a	296.61 ± 11.99 a	ND	68.33 a
*F. graminearum* GS2401 vs. *T. longibrachiatum* TL2501	ND	2.59 ± 0.29 b (↓48%)	91.27 ± 36.08 bc (↓69%)	ND	49.00 b (↓28%)
*F. graminearum* GS2401 vs. *T. koningiopsis* TK2502	ND	0.61 ± 0.06 b (↓88%)	46.98 ± 28.43 c (↓84%)	ND	46.83 b (↓31%)
*F. graminearum* GS2401 vs. *T. gamsii* TG2503	ND	0.85 ± 0.20 b (↓83%)	104.36 ± 7.94 b (↓65%)	ND	48.17 b (↓30%)
*F. verticillioides* GS2403 control	6.67 ± 0.93 a	4.35 ± 3.02 a	96.30 ± 20.88 a	0.49 ± 0.04 a	68.33 a
*F. verticillioides* GS2403 vs. *T. longibrachiatum* TL2501	0.51 ± 0.05 c (↓92%)	1.30 ± 0.29 b (↓70%)	15.13 ± 15.52 c (↓84%)	0.46 ± 0.03 a (↓6%)	31.00 c (↓55%)
*F. verticillioides* GS2403 vs. *T. koningiopsis* TK2502	1.73 ± 0.30 b (↓74%)	0.96 ± 0.21 b (↓78%)	70.13 ± 32.91 ab (↓27%)	0.21 ± 0.05 b (↓57%)	32.50 c (↓52%)
*F. verticillioides* GS2403 vs. *T. gamsii* TG2503	1.67 ± 0.30 b (↓75%)	1.05 ± 0.16 b (↓76%)	48.35 ± 7.70 bc (↓50%)	0.46 ± 0.20 a (↓6%)	36.50 b (↓47%)
*F. boothii* GS2407 control	ND	0.96 ± 0.31 a	58.89 ± 39.27 a	ND	57.17 a
*F. boothii* GS2407 vs. *T. longibrachiatum* TL2501	ND	ND c (↓100%)	32.36 ± 10.12 a (↓45%)	ND	22.83 b (↓60%)
*F. boothii* GS2407 vs. *T. koningiopsis* TK2502	ND	0.65 ± 0.03 ab (↓32%)	35.36 ± 7.98 a (↓40%)	ND	24.33 b (↓57%)
*F. boothii* GS2407 vs. *T. gamsii* TG2503	ND	0.51 ± 0.11 b (↓47%)	5.32 ± 11.79 a (↓90%)	ND	20.83 b (↓64%)
*F. subglutinans* GS2410 control	ND	ND	277.37 ± 121.07 a	0.33 ± 0.02 a	56.50 a
*F. subglutinans* GS2410 vs. *T. longibrachiatum* TL2501	ND	ND	53.43 ± 33.73 b (↓81%)	0.26 ± 0.01 a (↓21%)	18.33 b (↓68%)
*F. subglutinans* GS2410 vs. *T. koningiopsis* TK2502	ND	ND	246.21 ± 135.82 a (↓11%)	0.24 ± 0.08 a (↓27%)	16.00 b (↓72%)
*F. subglutinans* GS2410 vs. *T. gamsii* TG2503	ND	ND	46.88 ± 56.13 b (↓83%)	0.26 ± 0.06 a (↓21%)	19.50 b (↓65%)

Data in the table are means ± SD. Different lowercase letters indicate significant differences (*p* < 0.05) between the *Fusarium* control and its co-culture media, as determined by Duncan’s new multiple range test. ND, not detected. FUS, fumonisin; 3-AcDON, 3-acetyl-deoxynivalenol; ZEN, zearalenone; BEA, beauvericin. Downward arrows (↓) indicate percentage reductions in mycotoxin concentrations and mycelial growth of co-culture treatments relative to the pathogen-only control group.

## Data Availability

The original contributions presented in this study are included in the article. Further inquiries can be directed to the corresponding author.

## Abbreviations

The following abbreviations are used in this manuscript:

FUM	Fumonisin
3-AcDON	3-acetyl-deoxynivalenol
ZEN	Zearalenone
BEA	Beauvericin
ZOL	Zearalenol
